# Tecovirimat is active against various MPXV strains, while cidofovir, brincidofovir, trifluridine, and gemcitabine have no detectable MPXV-specific antiviral activity

**DOI:** 10.1016/j.virusres.2025.199615

**Published:** 2025-08-12

**Authors:** Nobuyo Higashi-Kuwata, Mariko Kato, Shin-ichiro Hattori, Yuki Takamatsu, Hiroaki Mitsuya

**Affiliations:** a Department of Refractory Viral Diseases, National Institute of Global Health and Medicine, Japan Institute for Health Security, Shinjuku-Ku, Tokyo 162-8655, Japan; b Kumamoto University Hospital, Kumamoto, 860-8556, Japan; c Experimental Retrovirology Section, National Cancer Institute, NIH, Bethesda, MD, USA

**Keywords:** Mpox, tecovirimat-resistant MPXV, antiviral agents, antiviral assays, morphometric assays

## Abstract

In treating patients with mpox, current treatment options are limited, with tecovirimat (TEC) being one of the few available. TEC has been approved by the European Medicines Agency (EMA) for treating patients with mpox and is in clinical use in Europe and Japan. However, following exposure to TEC, TEC-resistant variants such as A290V-containing variant (MPXV_R_^TEC/A290V^) emerge quickly. In such cases involving MPXV_R_^TEC/A290V^, alternative agents such as brincidofovir (BCV) have been used, although their efficacy remains controversial and their anti-MPXV activity is yet to be clearly defined. In the present work, we evaluated the anti-MPXV features of five agents (TEC; cidofovir, CDV; BCV; trifluridine, TFT; and gemcitabine, dFdC) reportedly active against various MPXV strains including MPXV^SPL2A7^, MPXV^Zr-599^, MPXV^Liberia^ and MPXV_R_^TEC/A290V^, employing cell-based quantitative assays using multiple target cell types such as VeroE6 cells as well as morphometric assays focusing on their cytostatic and cytotoxic natures. The EC_50_ values of TEC against MPXV^SPL2A7,^ MPXV^Zr-599^, and MPXV^Liberia^ were 0.001, 0.005, and 0.004 µM, respectively, without tangible cytotoxicity, while that against MPXV_R_^TEC/A290V^ was ∼130-fold greater with 0.13 µM. The EC_50_ values of CDV, BCV, TFT, and dFdC against MPXV_R_^TEC/A290V^ were 18, 1.8, 3.8, and 0.02 µM, respectively; however, the apparent anti-MPXV activity of these four agents was highly associated with their cytotoxicity as they were examined with qualitative and quantitative cell-based-morphometric assays. The data strongly show that none the four agents examined exhibited significant anti-MPXV activity and indicate that effective anti-MPXV agents active against wild-type and drug-resistant variants are urgently needed.

## Highlights

• Compounds reportedly active against MPXVs are in controversy and are revisited.
• Only tecovirimat among agents tested here exhibits MPXV-specific antiviral activity.
• Apparent anti-MPXV activity of agents except tecovirimat results from their toxicity.
• Novel agents more potent than tecovirimat are urgently needed for controlling mpox.

**Abbreviations:** MPXV, Monkeypox virus; ORF, Open Reading Frame; EC_50_, 50% effective concentration; CC_50_, 50% cytotoxicity concentration; qPCR, quantitative polymerase chain reaction

Monkeypox virus (MPXV), which belongs to the orthopoxvirus genus including smallpox virus, is the pathogenic virus that causes mpox (Lu et al, 2023; Mitjà *et al.*, 2023; Beiras *et al.*, 2024; Huang *et al.*, 2025). Historically, it was a zoonotic disease endemic in Central and West Africa, but in May 2022, European individuals who had not traveled to Africa were diagnosed to have the disease, and a global epidemic followed among homosexual men. Current treatment options for MPXV infection are limited with tecovirimat (TEC) being one of the few available (Frenois-Veyrat, *et al.*, 2022; Siegrist *et al.*, 2023). TEC has been approved by the European Medicines Agency (EMA) for treating patients with mpox and is in clinical use in Europe and Japan. However, following exposure to TEC, the emergence of tecovirimat-resistant MPXV variant (MPXV_R_^TEC^) has been reported (Smith *et al.*, 2023), posing a serious challenge in controlling mpox. Indeed, we have also isolated a TEC-resistant variant (MPXV/human/Japan/Tokyo/NCGM240303, GenBank: LC831698.1; hereafter referred as MPXV_R_^TEC/A290V^) strain harboring a A290V substitution in ORF F13L gene as previously reported by Lee’s group (Lee *et al.*, 2024). However, the features of anti-MPXV activity and safety of TEC and other agents previously reported to be active against MPXV such as cidofovir (CDV), brincidofovir (BCV) (a prodrug of CDV) (Moss *et al.*, 2024; Prévost *et al.*, 2024), trifluridine (TFT), and gemcitabine (dFdC) (Hishiki *et al.*, 2023) are not well-defined and remain to be determined. Under the current circumstances, clinical trial of brincidofovir is underway as part of “MpOx Study in Africain” (MOSA study. 2025) to study its safety and efficacy.

**Fig. 1:**
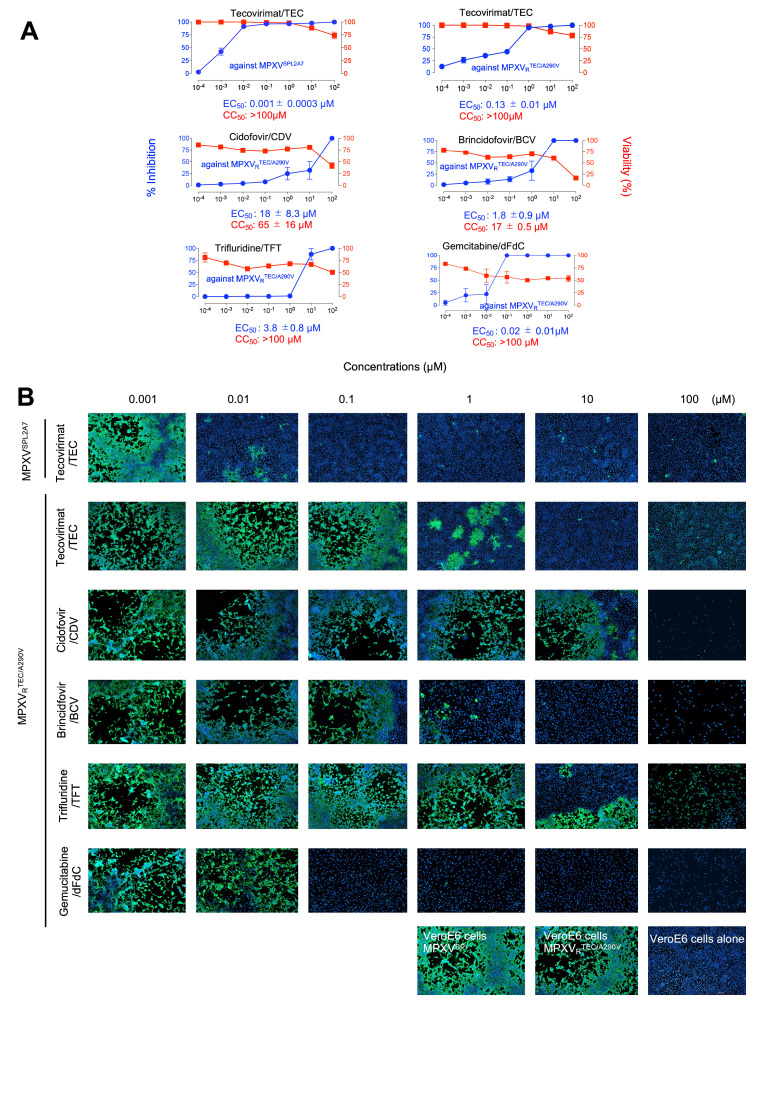
**Tecovirimat (TEC) exerts anti-MPXV activity against both wild-type and TEC-resistant MPXV clinical isolates**. VeroE6 cells were exposed to A290-wild-type or TEC-resistant A290V-carrring MPXV strain (MPXV^SPL2A7^ and MPXV_R_^TEC/A290V^, respectively) at 0.15 MOI for 3 hours, washed once with fresh medium, and cultured in the presence of each test compound for 5 days. Antiviral activity was evaluated using qPCR assay and immunocytochemistry, while cytotoxicity was evaluated with WST-8 assay and cell morphological observation. Panel **A** shows numerical antiviral and cell toxicity data and Panel **B** shows representative images of Immunocytochemistry. Data from three independent assays are shown as arithmetic means ± 1 S.D. from assays conducted on three independent occasions. As compound-free controls, vehicle alone samples for each compound were prepared. Before evaluation, we confirmed that cell culture with solvent ( 0.5% DMSO, 0.5% 0.1M NaOH, or 0.5% H_2_O) alone at the highest concentration of each dilution series showed no cytotoxicity. “VeroE6 cell alone” shows an image of cells treated with 0.5% 0.1M NaOH for BCV.

In the present study, we first determined the viral titers of Clade I-MPXV^Zr-599^, Clade IIa-MPXV^Liberia^, and two Clade IIb (MPXV^SPL2A7^ and MPXV_R_^TEC/A290V^) preparations (Andrei *et al.*, 2023) using the African green monkey’s kidney cell line, VeroE6, as previously described (Saijo *et al.*, 2006; Mills *et al.*, 2022; Hishiki *et al*., 2023) and evaluated anti-MPXV activity of TEC and other agents previously reported to be active against MPXV. To secure rigorous evaluation of anti-MPXV activity of compounds, cells were plated at relatively lower density (10,000 cells/well ) in 96-well microtiter culture plates, exposed to MPXV, replenished with fresh medium, exposed to test compounds, and cultured for 5 days. Details are described in Supplementary information (SI). As determined in the assays using VeroE6 cells, the EC_50_ values obtained with TEC were 0.005 ± 0.001, 0.004 ± 0.002, and 0.001 ± 0.002 µM against MPXVX^Zr-599^, MPXV^Liberia^ and MPXV^SPL2A7^, respectively, without tangible cytostaticity or cytotoxicity (Table 1 and Figure 1-A). However, the EC_50_ value of TEC against MPXV_R_^TEC/A290V^ (Clade IIb) was about 130-fold greater with 0.13 ± 0.01 µM than MPXV^SPL2A7^(Clade IIb). It was revealed in the present assay, all four test agents (CDV, BCV, TFT, and dFdC) apparently blocked the replication of MPXV_R_^TEC/A290V^ with EC_50_values of 18 ± 8.3, 1.8 ± 0.9, 3.8 ± 0.8 and 0.02 ± 0.01 µM, respectively; however, it was also apparent that the anti-MPXV activity of these four agents were associated with their cytostaticity and/or cytotoxicity. Therefore, we further asked whether the cytostaticity and/or cytotoxicity of the four agents were associated with the apparent anti-MPXV activity by employing immunocytochemistry. As seen in Figure 1-B, TEC effectively blocked the production of the virally-encoded proteins (stained in green) at 0.01 µM and above, when VeroE6 cells were exposed to MPXV^SPL2A7^, but no significant reduction in the number of the cells (seen as nuclei stained blue with DAPI) was seen even at 10 µM. When VeroE6 cells were exposed to MPXV_R_^TEC/A290V^, the viral protein production was blocked at 1 µM of TEC and above, largely in line with the observations in the qPCR assays (Figure 1-A). It was also noted that when cultured with CDV and BCV, the viral production was not well blocked even at 10 µM and 0.1 µM, respectively (shown in green), but the number of VeroE6 cells significantly decreased at 10 and/or 100 µM. TFT failed to block the viral protein production even at 10 µM and there was an apparently substantial reduction in the number of cells at 100 µM. dFdC also failed to block the viral protein production at 0.01 µM, but at 0.1 µM and above it appeared to have blocked the viral protein production but there were substantially fewer cells, and it was assumed that the observed apparent viral protein production inhibition was due to the cytostatic activity of dFdC (Figure 1-B).

**Table 1:** Anti-viral activity of tecovirimat and other agents against various MPXV strains.

	Against MPXV variants EC_50_ (µM)			
	MPXV^Zr-599^	MPXV^Liberia^	MPXV^SPL2A7^	MPXV_R_^TEC/A290V^
Compound	(Clade I)	(Clade IIa)	(Clade IIb)	(Clade IIb)
Tecovirimat	0.005 ± 0.001	0.004 ± 0.002	0.001 ± 0.002	0.13 ± 0.01
Cidofovir	17.3 ± 5.03	15.2 ± 3.02	12.2 ± 1.19	18 ± 8.3
Brincidofovir	2.13 ± 0.95	2.01 ± 0.85	2.03 ± 0.32	1.8 ± 0.9
Trifluridine	3.71 ± 1.45	3.48 ± 1.82	6.17 ± 1.26	3.8 ± 0.8
Gemcitabine	0.02 ± 0.02	0.01 ± 0.01	0.02 ± 0.02	0.02 ± 0.01

Anti-MPXV activity of compounds discussed in the present study was determined using qPCR of viral DNA with the lysates of cells and culture medium derived from MPXV-exposed VeroE6 cell culture. The amount of MPXV-DNA in each assay sample with a test compound was compared to no-compound control samples, and 50% effective concentration (EC_50_) was determined. Data from three independent assays are shown as arithmetic means ± 1 S.D.

**Fig. 2:**
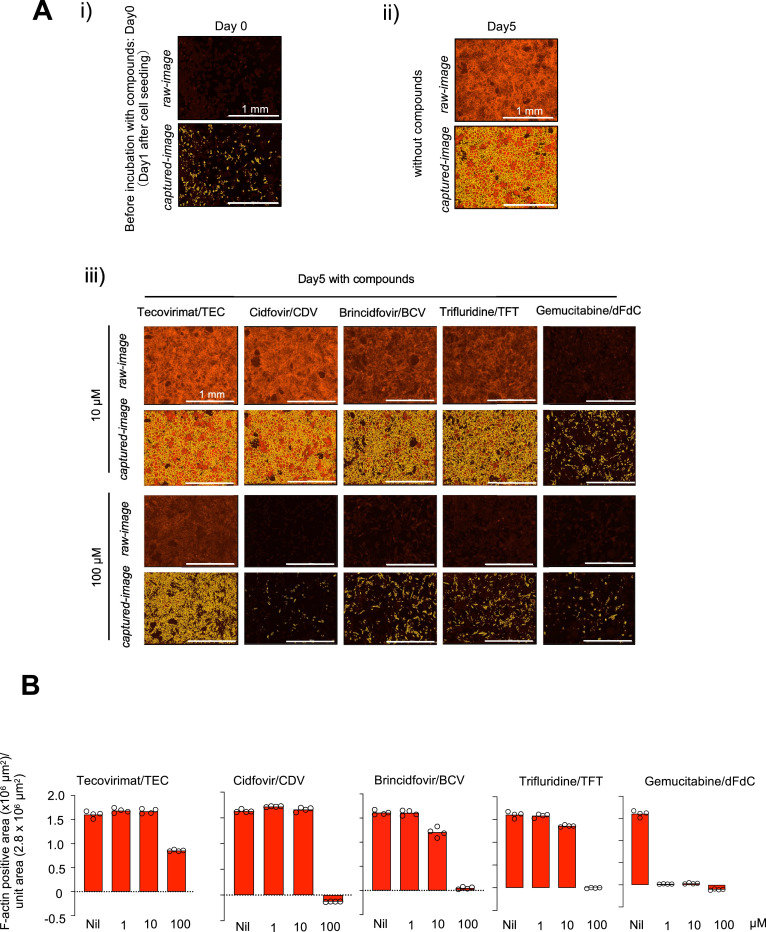
**Effects of various compounds on intracellular protein production : A**) Representative microscopic images used for measurement using Cytation 5 cell imaging multimode reader. (i) shows images before the addition of drugs, (ii) shows images of the no-compound control on day 5 in culture, and (iii) shows images on day 5 following the addition of each compound at 2 drug concentrations (10 and 100 µM). The top rows in (i) to (iii) panels show the images of F-actin (shown in red). “without compounds” denotes an image of cells treated with 0.5% 0.1M NaOH representing the compound-free controls. **B**) Summary of quantitative evaluation of F-actin production on Day 5 in culture with each compound using morphometric quantification of F-actin-positive signals. The quantity of F-actin produced was determined by capturing the signal intensity (shown in yellow). The signal intensity before drug exposure (on Day 0), was subtracted from the value on day 5. White bars in each figure in A denote the distance of 1 mm under the microscope. As compound-free controls, vehicle only samples for each compound were prepared. Nil means “solvent alone” for each compound. TEC, TFT, and dFdC are with 0.5% DMSO, CDV 0.5% H_2_O, and BCV 0.5% 0.1M NaOH.

Next, in an attempt to examine whether the observed apparent anti-MPXV activity described above was associated with the cytostaticity/cytotoxicity inherent to test compounds, we conducted quantitative morphometric assay using the image capturing function of Cytation 5 cell imaging multimode reader (Galenkamp *et al.*, 2021) as previously described (Takamatsu *et al.*, 2023; Higashi-Kuwata *et al.*, 2025). To perform a rigorous evaluation of the cytostaticity/cytotoxicity of compounds, we utilized the same cell line, Vero E6, and same cell density used in the above antiviral assays. The upper panel in Figure 2-A-i shows a raw image of VeroE6 cells without capturing (*i.e.*, “masking”) under Cytation 5, while the lower panel in Figure 2-A-i shows the image with capturing the F-actin within the cells (highlighted in yellow), which represents cellular cytoskeleton filamentous actin and is visualized with Texas Red-labeled phalloidin that binds to F-actin. The presence of substantial amounts of cellular F-actin shows that the cells have good protein production ability. On day 5 in culture, robust mesh-like F-actin is seen in a greater amount in the upper panel in Figure 2A-ii (control image), indicating the cells have been healthy and robustly proliferated. When VeroE6 cells were cultured in the continuous presence of 10 µM TEC over 5 days, the amount of F-actin appeared virtually the same as that in VeroE6 cells without test compound seen in Figure 2-A-ii; while 100 µM TEC only moderately decreased the amount of F-actin. When VeroE6 cells were cultured in the presence of 10 µM CDV, there was no significant decrease in the amount of F-actin as compared with that in the control image. However, co-culturing the cells with 100 µM CDV, the amount of F-actin was greatly reduced. When the cells were cultured with each of BCV or TFT, the amounts of F-actin also markedly reduced at 100 µM. In the case of dFdC, the amounts of F-actin were greatly reduced at 10 and 100 µM (Figure 2-A-iii). Figure 2-B summarizes the results of quantitative evaluation of F-actin production by VeroE6 cells cultured for 5 days with each compound using morphometric quantification of F-actin-positive areas as compared with the F-actin amounts on Day 0. The data clearly show that TEC at 100 µM suppressed the protein production by VeroE6 cells by about 50%, while three compounds (CDV, BCV, and TFT) virtually totally blocked the protein production at 100 µM. Notably, dFdC, in the presence of all concentrations tested (1, 10, and 100 µM), totally blocked the cells’ protein production. These data, together, strongly suggested that the apparent anti-MPXV activity of CDV, BCV, and TFT, and in particular, dFdC was strongly associated with the reduced protein production caused by such agents.

**Fig. 3:**
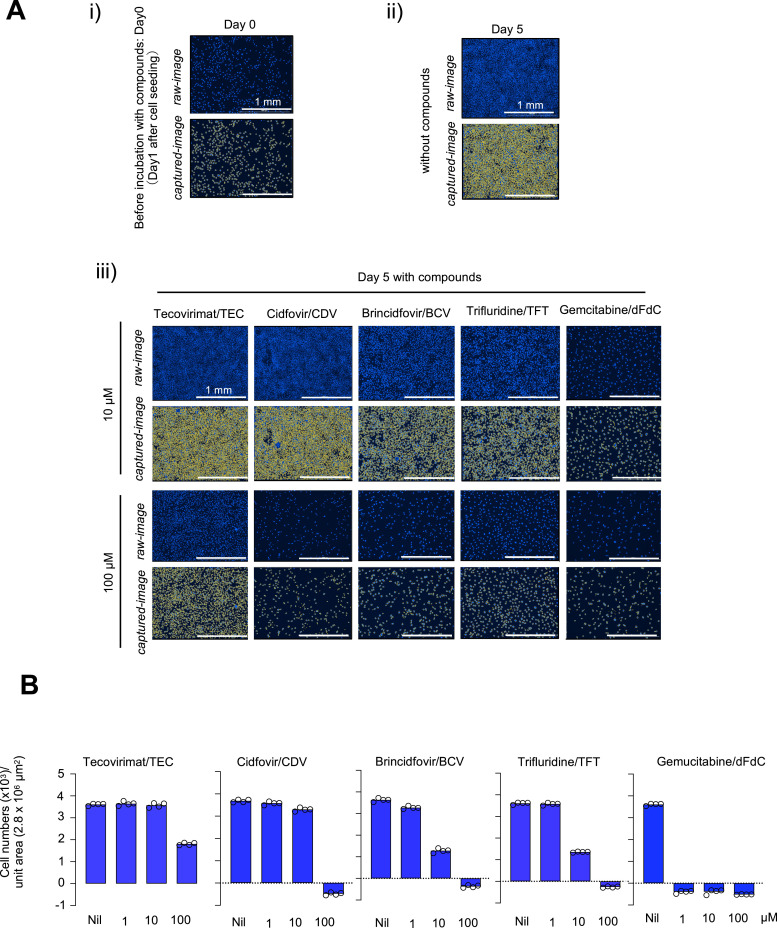
**Effects of various compounds on cell proliferation: A**) Representative microscopic images used for measurement using Cytation 5 cell imaging multimode reader. (i) shows images before the addition of drugs, (ii) shows images of the no-compound control on day 5 in culture, and (iii) shows images on day 5 following the addition of each compound at 2 drug concentrations (10 and 100 µM). The top rows in (i) to (iii) panels show the images of nuclei (shown in blue). “without compounds” denotes an image of cells treated with 0.5% 0.1M NaOH representing the compound-free controls. **B**) Summary of quantitative evaluation of cell counts on Day 5 in culture with each compound using morphometric quantification of DAPI-positive signals. The quantity of nuclei number was determined by capturing the signal intensity (shown in yellow). The signal intensity before drug exposure (on Day 0), was subtracted from the value on day 5. White bars in each figure in A denote the distance of 1 mm under the microscope. As compound-free controls, vehicle only samples for each compound were prepared. Nil means the solvent of each compound. TEC, TFT, and dFdC are with 0.5% DMSO, CDV 0.5% H_2_O, and BCV 0.5% 0.1M NaOH.

Since it was suggested that the apparent anti-viral activity seen with CDV, BCV, TFT, and dFdC was associated with their inhibitory effects on the cellular protein production, we also asked whether the cellular growth was affected by such agents by counting the cell number using DAPI staining and the image capturing function of Cytation 5 cell imaging multimode reader (Figure 3). As shown in Figure 3-A-Fig. 3, Fig. 3-B, 10 µM TEC did not reduce the number of cells following the 5-day culture although 100 µM TEC caused moderate reduction. CDV did not appear to reduce the cell number at 10 µM, but 100 µM CDV caused marked reduction. BCV and TFT showed moderate levels of cellular reduction at 10 µM, but both caused marked reduction in the number of cells at 100 µM. As shown in Figure 3-B, when the cell number on day 5 in culture was subtracted with the cell number before the addition of compound (Day 0), the resulting values for CDV, BCV, and TFT at 100 µM were all below zero (Figure 3-B). Notably, when the cells were cultured even in the presence of as low as 1 µM, dFdC showed significant reduction in the cell number. The marked levels of reduction in the number of cells seen with the four compounds are highly likely to represent their cytocidal effects. These data together with the inhibition of cellular protein production strongly suggest that such cytotoxic, and cytocidal natures resulted in the apparent anti-MPXV activity.

The four compounds, CDV, BCV, TFT, and dFdC, determined not to have specific anti-MPXV activity as mentioned above, are in the category of nucleoside analogs. A large body of literature has shown that most nucleoside analogs have to be anabolically tri-phosphorylated to exert their biological and/or pharmaceutical activity; and therefore, such activity may or may not be seen depending on species (Dahlberg *et al.*, 1987), cell types (Hutching *et al.*, 2019), and activation states (Gao *et al.*, 1993; Gao *et al.*, 1994). Thus, in addition to the African green monkey’s kidney cell line (VeroE6), we employed two additional human cell lines, a hepatocytic cancer cell line (Huh7) and a cervical cancer cell line (HeLa) and examined whether the nucleoside analogs, CDV, that has been shown to have ani-MPXV activity in limited studies in monkeys and humans and has been recommended by US-CDC for the compassionate use for patients with severe mpox (CDC, 2025) (an active form of BCV) and BCV exert anti MPXV activity.

**Fig. 4:**
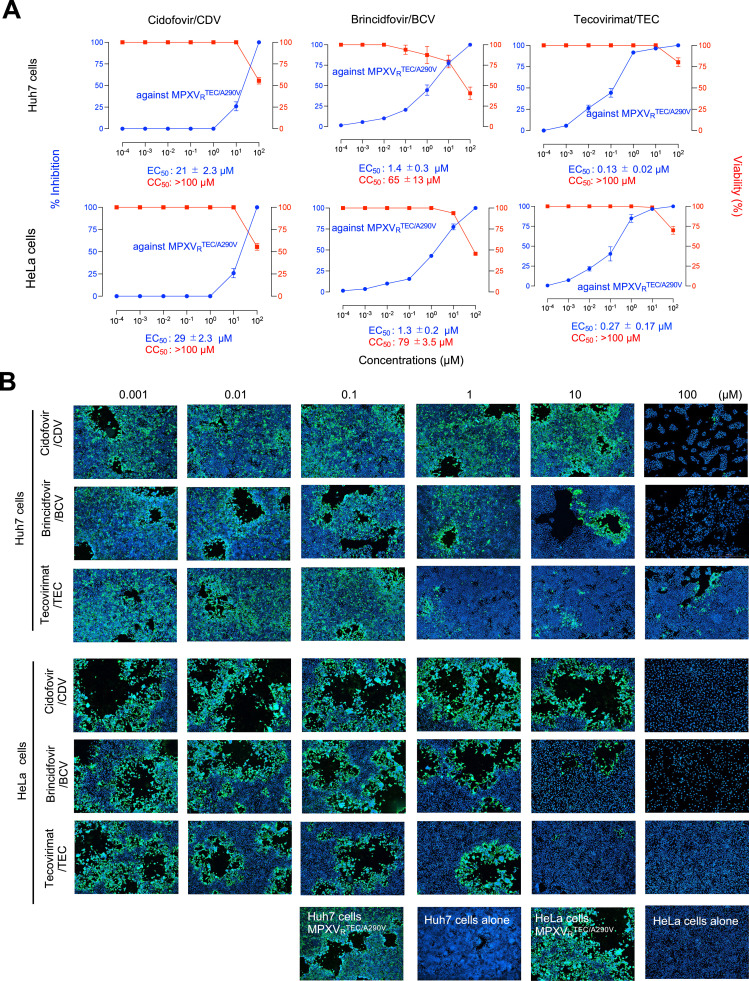
**Cidofovir and Brincidofovir fail to show anti-MPXV activity in two human cells.** Huh7 and HeLa cells were exposed to TEC-resistant MPXV strain (MPXV_R_^TEC/A290V^) at 0.15 MOI for 3 h, washed once with fresh medium, and cultured with cidofovir or TEC for 5 days. Antiviral activity was evaluated using qPCR and cell immunostaining, and cytotoxicity was evaluated with WST-8 assay and cell morphological observation. Panel **A** shows summarized data and Panel **B** indicates representative images of Immunocytochemistry. Data from three independent assays are shown as arithmetic means ± 1 S.D. As compound-free controls, vehicle only samples were prepared. Before evaluation, we confirmed that cell culture with the solvents ( 0.5% DMSO, 0.5% 0.1M NaOH, or 0.5%H_2_O) alone at the highest concentration of each dilution series induced no cytotoxicity. “Huh7 cells alone” and “HeLa cells alone” show images of cells treated with 0.5% 0.1M NaOH for BCV.

When the anti-MPXV activity of CDV against MPXV_R_^TEC/A290V^ was determined in Huh7 and HeLa cells, it failed to block the replication of MPXV even at 10 µM with its EC_50_ values of 21 ± 2.3 µM and 29 ± 2.3 µM, respectively, and at 100 µM the agent showed cytotoxicity, reducing the number of cells. In the smiler way, when the anti-MPXV activity of BCV against MPXV_R_^TEC/A290V^ was determined in Huh7 and HeLa cells, it failed to block the replication of MPXV at 1 µM with its EC_50_ values of 1.4 ± 0.3 µM and 1.3 ± 0.2 µM, respectively, and at 10-100 µM the agent showed cytotoxicity, reducing the number of cells. Meanwhile, TEC showed substantial anti-MPXV activity with its EC_50_ values of 0.13 ± 0.02 µM and 0.27 ± 0.17 µM, respectively, without cytostaticity and/or cytotoxicity (Figure 4). These data, taken together, showed that CDV and BCV failed to exert its anti-MPXV activity in two human cells as well.

The present work strongly suggests that the previously reported anti-MPXV activity of CDV, BCV, TFT, and dFdC result from their cytotoxicity and the apparent reduction of viral production by each of them was thought to be associated with their anti-MPXV activity. Regarding BCV, the MOSA study (Enrollment starts in Africa CDC-LED Mpox therapeutic study, 2025.) was designed to administer BCV once a week for two weeks and to determine toxicity at the systemic level, which might be controlled by monitoring liver and kidney function. Although it is possible to detect apparent antiviral effect at the systemic level through mechanisms such as eliminating/sterilizing infected cells, one cannot be certain that such apparent effect resulted from virus-specific antiviral activity. The present data also elucidated that only TEC among the agents examined exerts salient MPXV-specific activity. Nevertheless, no significant efficacy of TEC in the recently conducted international placebo-controlled clinical trial (the Study of Tecovirimat for Mpox: STOMP_Tecovirimat was safe but did not improve mpox resolution or pain, 2024) was seen among adults with clade II mpox. Another trial PALM007 operated in the Democratic Republic of the Congo also showed that TEC failed to shorten the duration of mpox lesions in treating clade I mpox among children and adults (The antiviral tecovirimat is safe but did not improve clade I mpox resolution in Democratic Republic of the Congo, 2024). In this regard, the major and critical force for recovery from MPXV-infection is the host's immune response largely as follows: (i) first line of defense that is activated shortly after infection, involving natural killer cells, macrophages, and interferon release *etc.*, helping contain the virus and signaling the following adaptive immune response (Lum *et al.*, 2022) ; and (ii) the adaptive immune response (main recovery driver) kicks in involving T cells crucial for killing infected cells, MPXV-neutralizing-antibodies-producing B cells, and long-term-immunity-conferring memory T cells.

The reason why TEC failed to show significant efficacy in the rigorous STOMP clinical trial is assumed that most MPXV-infected patients enrolled were with mild to moderate mpox and a low risk of developing severe diseases. Therefore, those enrollees duly recovered with proper immunity in action, and only moderately potent TEC treatment failed to make appreciable difference. It is noteworthy, however, that the PALM007 study had 1.7% overall mortality among enrollees regardless of whether they received TEC or not, which was much lower than the DRC’s national average mpox mortality of 3.6%, also suggesting that TEC did not make significant difference.

Another possible reason for the TEC failure is that TEC was administered too late since patients with at least one mpox skin lesion and positive polymerase-chain-reaction results for clade I MPXV were assigned in a 1:1 ratio to receive tecovirimat or placebo in this study (PALM007 Writing Group, 2025). Those clinical trial data, albeit yet preliminary and still underway, together with our present data that only TEC exerted MPXV-specific activity but not others, strongly suggest that anti-MPXV agents that exhibit greater barrier to resistance and/or a different mode(s) of actions is urgently needed for controlling mpox.

All experiments with MPXV strains were approved by the President of National Institute of Global Health and Medicine, and Japanese Government, following consideration by the Institutional Committee (approval ID: 2023-M058) and were carried out in accordance with relevant guidelines in biosafety level three (BSL3) facility at the Japan Institute for Health Security.

## Web references

Enrollment Starts in Africa CDC-LED Mpox Therapeutic Study (MOSA), 2025. https://africacdc.org/news-item/enrollment-starts-in-africa-cdc-led-mpox-therapeutic-study-mosa/ (accessed 5 July 2025)

MOSA study, 2025. https://mpx-response.eu/studies/ (accessed 5 July 2025)

Tecovirimat Was Safe but Did Not Improve Mpox Resolution or Pain, 2024.

https://www.nih.gov/news-events/news-releases/nih-study-finds-tecovirimat-was-safe-did-not-improve-mpox-resolution-or-pain (accessed 5 July 2025)

The antiviral tecovirimat is safe but did not improve clade I mpox resolution in Democratic Republic of the Congo, 2024. https://www.nih.gov/news-events/news-releases/antiviral-tecovirimat-safe-did-not-improve-clade-i-mpox-resolution-democratic-republic-congo (accessed 5 July 2025)

## CRediT authorship contribution statement

**Nobuyo Higashi-Kuwata:** Writing – review & editing, Writing – original draft, Visualization, Validation, Resources, Project administration, Methodology, Investigation, Funding acquisition, Formal analysis, Data curation, Conceptualization. **Mariko Kato:** Investigation, Formal analysis. **Shin-ichiro Hattori:** Investigation. **Yuki Takamatsu:** Investigation, Funding acquisition. **Hiroaki Mitsuya:** Writing – review & editing, Supervision, Project administration, Funding acquisition, Data curation, Conceptualization.

## Declaration of competing interest

The authors declare that they have no known competing financial interests or personal relationships that could have appeared to influence the work reported in this paper.

## Data Availability

Data will be made available on request.

## Appendix Supplementary materials

Image, application 1
