# Supplementary material for: Tecovirimat is active against various MPXV strains, while cidofovir, brincidofovir, trifluridine, and gemcitabine have no detectable MPXV-specific antiviral activity
Source: Virus Res. 2025 Aug 12;360:199615. doi: 10.1016/j.virusres.2025.199615 (PMC12396559; doi:10.1016/j.virusres.2025.199615)
Supplement: Supplementary file 1 [file mmc1.docx]

**Tecovirimat Is Active against Various MPXV Strains, while Cidofovir, Brincidofovir, Trifluridine, and Gemcitabine Have No Detectable MPXV-Specific Antiviral Activity**

Nobuyo^*^ Higashi-Kuwata^a^, Mariko Kato^a^, Shin-ichiro Hattori^a^, Yuki Takamatsu^a^,

Hiroaki Mitsuya^a,b,c*^

*^a^Department of Refractory Viral Diseases, National Institute of Global Health and Medicine, Japan Institute for Health Security, Shinjuku-ku, Tokyo 162-8655, Japan; ^b^Kumamoto University Hospital, Kumamoto, 860-8556, Japan*

*^c^Experimental Retrovirology Section, National Cancer Institute, NIH, Bethesda, MD, USA*

***Contact information of the corresponding authors:**

**Hiroaki Mitsuya, M.D., Ph.D.**

Experimental Retrovirology Section

HIV and AIDS Malignancy Branch

National Cancer Institute, NIH, Bethesda, MD 20892, USA

Phone: +1-240-858-3260, Email: [hiroaki.mitsuya2@nih.gov](mailto:hiroaki.mitsuya2@nih.gov)

Department of Refractory Viral Diseases,

National Institute of Global Health and Medicine,

Japan Institute for Health Security

1-21-1 Toyama, Shinjuku-ku, Tokyo, 162-8655, Japan

Phone : +81-33-202-7181

Fax : +81-33-202-7364, Email: mitsuya.h@jihs.go.jp

**Nobuyo Higashi-Kuwata, D.V.M., Ph.D.**

National Institute of Global Health and Medicine,

Japan Institute for Health Security

1-21-1 Toyama, Shinjuku-ku, Tokyo, 162-8655, Japan

Phone : +81-33-202-7181

Fax : +81-33-202-7364

Email : kuwata.n@jihs.go.jp

**MATERIALS AND METHODS**

**Cells**

An African green monkey kidney-derived cell line (VeroE6) cells were obtained from the American Type Culture Collection (ATCC) (CRL-1586) (Manassas, VA) and were maintained in Dulbecco’s modified Eagle’s medium (d-MEM) supplemented with 10% fetal bovine serum (FCS), 100 μg/ml of penicillin, and 100 μg/ml of streptomycin. A human cervical cancer cell line HeLa cells were obtained from the Japanese Collection of Research Bioresources (JCRB) Cell Bank (JCRB9004, Osaka, Japan) and were maintained the same conditioned medium as that VeroE6 cell line. A human hepatoma cell line (Huh7 cells) is a kind gift from Dr. T. Wakita of National Institute of Infectious Diseases in Japan and cultured in DMEM supplemented with 10% FCS. All cells were maintained at 37 ºC with 5% CO_2_. The cells were regularly tested and confirmed to be negative for mycoplasma contamination by using PCR.

**Virus preparation**

Monkeypox virus strain MPXV^SPL2A7^ (Clade IIb), MPXV^Liberia^ (Clade IIa), and MPXV^Zr-599^ (Clade I) was obtained from the National Institute of Infectious Diseases (Tokyo, Japan). A MPXV/human/Japan/Tokyo/NCGM240303 (GenBank: LC831698.1)(Clade IIb) strain caring a A290V mutation which reportedly confers tecovirimat (TEC)-resistance (Lee et al, 2024) was isolated our facility and hereafter is referred to as MPXV_R_^TECA290V^. The viral titer was determined by plaque assay using VeroE6 cells, as previously described (Saijo et al., 2006). Virus stocks were stored at −80°C until use.

**Preparation of compounds**

Tecovirimat (TEC) and trifuluridine (TFT) were purchased from Selleck Biotechnology (Yokohama, Kanagawa, Japan), while bincidofovir (BCV), cidfovir (CDV), and gemcitabine (dFdC) were from Med Chem Express (Monmouth Junction, NJ, USA). According to the manufacturer's data sheets, the compounds were dissolved in appropriate solvents. Each solvent and stock solution concentration, including product # for each compound in this study, is shown in Table S1.

**Antiviral activity and cytotoxicity assays**

We first determined the viral titers of Clade I-MPXV^Zr-599^, Clade IIa-MPXV^Liberia^, and two Clade IIb (MPXV^SPL2A7^ and MPXV_R_^TEC/A290V^) preparations using the African green monkey’s kidney cell line, VeroE6, as previously described (Saijo et al., 2006; Mills et al., 2022; Hishiki et al, 2023). For general cell-based antiviral assay, VeroE6, Huh7, or HeLa cells were seeded in a 96-well plate (1 × 10^4^ cells/ well) and incubated for one day, then the virus was inoculated into the culture at each multiplicity of infection (MOI): MPXV^SPL2A7^, 0.15; MPXV^Liberia^, 0.15; MPXV^Zr-599^, 0.15; MPXV_R_^TEC/A290V^, 0.15. The MOI values were determined by plaque assay using VeroE6 cells. Three hours post exposure, the virus was removed and the cells were washed out one time with culture medium and incubated for 5 days with each drug solution. Each compound solvent was placed for compound-free controls, as shown in Table S1. In brief, 0.5% DMSO, 0.5% 0.1M NaOH, or 0.5% H_2_O was used as the start dilution solutions for the vehicles alone samples. Each vehicle alone/solvent solution was serially diluted in the same manner as compound solution on the same plate to serve as compound-free controls. Following the incubation, culture supernatants and cells were harvested and viral DNA was extracted using a QIAamp DNA minikit (Qiagen, Hilden, Germany), and quantitative qPCR was then performed using Premix Ex Taq™ (Probe qPCR) (TaKaRa Bio, Shiga, Japan) and a 7500 Fast Real-Time PCR Instrument (Applied Biosystems, Waltham, MA, USA) following the instructions of the manufacturers. The primers and probe targeting the loci for MPXV-F3L were 5’- CATCTATTATAGCATCAGCATCAGA -3’ (forward), 5’- GATACTCCTCCTCGTTGGTCTAC -3’ (reverse), and 5’-FAM- TGT AGG CCG TGT ATC AGCATC CATT-black hole quencher 1 (MGBEQ1)-3’ (probe) (Mills et al, 2022) The amount of MPXV-DNA in each assay sample with a test compound was compared to no-compound control samples, and 50% effective concentration (EC_50_) was determined using the Forecast function of Microsoft Excel software. All experiments with MPXV strains were approved by the President of National Institute of Global Health and Medicine, and Japanese Government, following consideration by the Institutional Committee (approval ID: 2023-M058) and were carried out in accordance with relevant guidelines in biosafety level three (BSL3) facility at the Japan Institute for Health Security.

To determine the cytotoxicity of each compound, cells were seeded in a 96-well plate (1 ×10^4^ cells/well). One day later, various concentrations of each compound were added, and cells were incubated for additional 5 days. The 50% cytotoxic concentrations (CC_50_) values were determined using the WST-8 assay and Cell Counting Kit-8 (Dojindo, Kumamoto, Japan). Data from three independent assays are shown as arithmetic means ± 1 S.D.

**Immunocytochemistry**

Cells in a 96-well microtiter culture plate were fixed with 4% paraformaldehyde–phosphate-buffered saline (PBS) for 15 min, washed with PBS (300 μL/well) three times for 5 min each time, and then blocked with a blocking buffer (10% goat serum, 1% bovine serum albumin [BSA], 0.3% Triton X-100, PBS 1x) for 1h. After removal of the blocking buffer, the cells were immediately stained with a Rabbit Anti-Vaccinia Virus polyclonal antibody (Abcam) overnight at 4 °C (Hishiki et al, 2023). The stained cells were washed with PBS (300 μL/well) three times for 5 min each time, and the cells were incubated with secondary antibody Goat Anti-Rabbit IgG H&L polyclonal antibody (Alexa Fluor® 488) (Abcam), DAPI (4’,6-diamidino-2-phenylindole) solution (Thermo Fisher Scientific)-PBS (50 μL/well) was added to stain nuclei. Signals were acquired with a Cytation 5 cell imaging multi-mode reader (BioTek, Winooski, VT, USA).

For qualitative evaluation of cell toxicity caused by drugs, morphometrical analysis was also performed. VeroE6 cells were seeded in a 96-well plate (1 × 10^4^ cells/ well) and incubated for one day, then incubated for 5 days with each drug solution. On day5, cells were fixed with 4% paraformaldehyde–phosphate-buffered saline (PBS) for 15 min, washed with PBS (300 μL/well) three times for 5 min each time, and then blocked with a blocking buffer (10% goat serum, 1% bovine serum albumin [BSA], 0.3% Triton X-100, PBS 1x) for 1h. After removal of the blocking buffer, the cells were immediately stained with a normal Rabbit polyclonal IgG, isotype control (Abcam) overnight at 4 °C. The stained cells were washed with PBS (300 μL/well) three times for 5 min each time, and the cells were incubated with secondary antibody Goat Anti-Rabbit IgG H&L polyclonal antibody (Alexa Fluor® 488) (Abcam), together with Texas Red-X dye-conjugated phalloidin (Thermo Fisher Scientific) for F-actin visualization for 2 h and DAPI for nuclei staining. Images of F-actin were taken setting at Excitation 568/Emission 647 under following conditions illumination intensity: 10, integration time: 2344, and camera gain 10, while that of nuclei were captured setting at Excitation 377/Emission 477 under following conditions, illumination intensity: 10, integration time: 334, and camera gain: 11. The number of positively stained area in red (F-actin)(threshold of > 6000 pixcls) and in blue (cell nuclei) (threshold of >5000 pixcls) was measure or counted on three-four randomly selected areas in 40-fold magnification using Gen5 software (BioTek Instruments) (Galenkamp et al, 2021) and as previously described (Takamatsu et al,2023; Higashi-Kuwata et al, 2025).

**References**

**Galenkamp** KMO, Galapate CM, Zhang Y, Commisso C. Automated Imaging and Analysis for the Quantification of Fluorescently Labeled Macropinosomes. J Vis Exp. 2021 Aug 24;(174):10.3791/62828. doi: 10.3791/62828. PMID: 34515683; PMCID: PMC8919987.

**Higashi-Kuwata N**, Bulut H, Hayashi H, Tsuji K, Ogata-Aoki H, Kiso M, Takamune N, Kishimoto N, Hattori SI, Ishii T, Kobayakawa T, Nakano K, Shimizu Y, Das D, Saruwatari J, Hasegawa K, Murayama K, Sukenaga Y, Takamatsu Y, Yoshimura K, Aoki M, Furusawa Y, Okamura T, Yamayoshi S, Kawaoka Y, Misumi S, Tamamura H, Mitsuya H. An orally available P1'-5-fluorinated Mpro inhibitor blocks SARS-CoV-2 replication without booster and exhibits high genetic barrier. PNAS Nexus. 2025 Jan 7;4(1):pgae578. doi: 10.1093/pnasnexus/pgae578. PMID: 39831159; PMCID: PMC11740726.

**Hishik**i T, Morita T, Akazawa D, Ohashi H, Park ES, Kataoka M, Mifune J, Shionoya K, Tsuchimoto K, Ojima S, Azam AH, Nakajima S, Kawahara M, Yoshikawa T, Shimojima M, Kiga K, Maeda K, Suzuki T, Ebihara H, Takahashi Y, Watashi K. Identification of IMP Dehydrogenase as a Potential Target for Anti-Mpox Virus Agents. Microbiol Spectr. 2023 Aug 17;11(4):e0056623. doi: 10.1128/spectrum.00566-23. Epub 2023 Jul 6. PMID: 37409948; PMCID: PMC10434032.

**Lee** M, Choi CH, Kim JW, Sim G, Lee SE, Shin H, Lee JH, Choi MM, Yi H, Chung YS. Prolonged viral shedding in an immunocompromised Korean patient infected with hMPXV, sub-lineage B.1.3, with acquired drug resistant mutations during tecovirimat treatment. J Med Virol. 2024 Mar;96(3):e29536. doi: 10.1002/jmv.29536. PMID: 38488495.

**Mills** MG, Juergens KB, Gov JP, McCormick CJ, Sampoleo R, Kachikis A, Amory JK, Fang FC, Pérez-Osorio AC, Lieberman NAP, Greninger AL. Evaluation and clinical validation of monkeypox (mpox) virus real-time PCR assays. J Clin Virol. 2023 Feb;159:105373. doi: 10.1016/j.jcv.2022.105373. Epub 2022 Dec 23. PMID: 36603329; PMCID: PMC9783225.

**Takamatsu Y**, Hayashi S, Kumamoto H, Imoto S, Tanaka Y, Mitsuya H, Higashi-Kuwata N. A novel anti-HBV agent, E-CFCP, restores Hepatitis B virus (HBV)-induced senescence-associated cellular marker perturbation in human hepatocytes. Virus Res. 2023 May;329:199094. doi: 10.1016/j.virusres.2023.199094. Epub 2023 Mar 23. PMID: 36933835; PMCID: PMC10194405.

**Saijo** M, Ami Y, Suzaki Y, Nagata N, Iwata N, Hasegawa H, Ogata M, Fukushi S, Mizutani T, Sata T, Kurata T, Kurane I, Morikawa S. LC16m8, a highly attenuated vaccinia virus vaccine lacking expression of the membrane protein B5R, protects monkeys from monkeypox. J Virol. 2006 Jun;80(11):5179-88. doi: 10.1128/JVI.02642-05. PMID: 16698998; PMCID: PMC1472157.

**Table S1. A list of compound preparation**

| Compounds | Solvent | Concentration | | Supplier  Product#, purity  CAS No. |
| --- | --- | --- | --- | --- |
|  |  | Stock solution in each solvent | Starting solution  in cell culture medium |  |
| TEC  (tecovirimat) | DMSO  (dimethyl sulfoxide) | 20mM | 100µM (200) | Selleck  S3380, 99.86%  869572-92-9 |
| CDV  (cidfovir) | H_2_O | 20mM | 100µM (200) | MedchemExpress  HY-17438, 99.95%  113852-37-2 |
| BCV  (bincidofovir) | 0.1M NaOH | 20mM | 100µM (200) | MedchemExpress  HY-14532, 99.46%  444805-28-1 |
| TFT  (trifuluridine) | DMSO  (dimethyl sulfoxide) | 20mM | 100µM (200) | Selleck  S1778, 99.96%  70-00-8 |
| dFdC  (gemcitabine) | DMSO  (dimethyl sulfoxide) | 20mM | 100µM (200) | MedchemExpress  HY-17026, 99.92%  95058-81-4 |

Each stock compound solution was serially diluted in the same manner on the same plate as compound-free controls were prepared. The numbers in parentheses indicate the dilution ratio from the stock solution. Before evaluation, we confirmed that cell culture with the solvents (0.5% DMSO, 0.5% 0.1M NaOH, or 0.5% H_2_O) alone at the highest concentration of each dilution series had no cytotoxicity.
